# Age at antiretroviral therapy initiation and cell-associated HIV-1 DNA levels in HIV-1-infected children

**DOI:** 10.1371/journal.pone.0195514

**Published:** 2018-04-12

**Authors:** Louise Kuhn, Maria Paximadis, Bianca Da Costa Dias, Shayne Loubser, Renate Strehlau, Faeezah Patel, Stephanie Shiau, Ashraf Coovadia, Elaine J. Abrams, Caroline T. Tiemessen

**Affiliations:** 1 Gertrude H. Sergievsky Center, College of Physicians and Surgeons, Columbia University, New York, New York; 2 Department of Epidemiology, Mailman School of Public Health, Columbia University, New York, New York; 3 Empilweni Services and Research Unit, Rahima Moosa Mother and Child Hospital, Department of Paediatrics and Child Health, Faculty of Health Sciences, University of the Witwatersrand, Johannesburg, South Africa; 4 Centre for HIV and STIs, National Institute for Communicable Diseases, Johannesburg, South Africa; 5 Faculty of Health Sciences, University of the Witwatersrand, Johannesburg, South Africa; 6 ICAP at Columbia, Mailman School of Public Health and Department of Pediatrics, College of Physicians & Surgeons, Columbia University, New York, New York, United States of America; University of Pittsburgh, UNITED STATES

## Abstract

**Background:**

The latent viral reservoir is the major obstacle to achieving HIV remission and necessitates life-long antiretroviral therapy (ART) for HIV-infected individuals. Studies in adults and children have found that initiating ART soon after infection is associated with a reduction in the size of the HIV-1 reservoir. Here we quantified cell-associated HIV-1 DNA in early-treated but currently older HIV-infected children suppressed on ART.

**Methods:**

The study participants comprised of a cohort of 146 early-treated children with HIV-1 RNA <50 copies/ml enrolled as part of a clinical trial in Johannesburg, South Africa. A stored buffy coat sample collected after a median 4.3 years on ART and where HIV-1 RNA was <50 copies/ml was tested for cell-associated HIV-1 DNA levels. An in-house, semi-nested real-time quantitative hydrolysis probe PCR assay to detect total HIV-1 subtype C proviral DNA was used. Children were followed prospectively for up to 3 years after this measurement to investigate subsequent HIV-1 RNA rebound/failure while remaining on ART. Age at ART initiation, HIV-1 RNA decline prior to HIV-1 DNA measurement and other factors were investigated.

**Results:**

A gradient between age at ART initiation and later HIV-1 DNA levels was observed. When ART was started <2 months of age, the lowest levels of cell-associated HIV-1 DNA (median 1.4 log_10_copies/10^6^ cells, interquartile range [IQR] 0.95–1.55) were observed compared to ART started at 2–4 months (median 1.68, IQR 1.26–1.97) or 5–14 months of age (median1.98, IQR 1.69–2.25). A low CD4 T-cell count pre-treatment predicted higher levels of HIV-1 DNA on later testing. The probability of HIV-1 RNA rebound >50 copies/ml whilst on ART within 3 years after the DNA measurement was 2.07 (95% CI: 1.352–3.167) times greater if the HIV-1 DNA level was above the median of 55 copies/10^6^ cells.

**Conclusions:**

Cell-associated HIV-1 DNA levels measured after more than 4 years on ART were lower the younger the age of the child when ART was initiated. This marker of the size of the viral reservoir also predicted subsequent viral rebound/treatment failure while ART was sustained. The results provide additional evidence of the benefits of prompt diagnosis and early ART initiation in newborns and infants.

## Introduction

The latent viral reservoir is the major obstacle to achieving HIV remission and necessitates life-long antiretroviral therapy (ART) for HIV-infected individuals. Studies have shown that the size of the HIV-1 reservoir is reduced in adults identified soon after primary infection and started rapidly on ART compared to those initiated on ART during chronic infection [1–3]. A minority (5–15%) of adults treated during primary infection have been observed to control viremia after ART is withdrawn [4–8]. Generally for adults who initiate ART during chronic infection, almost all have immediate viral rebound when ART is withdrawn [4–8].

For perinatally-infected children, age at ART Initiation is roughly equivalent to time since infection. Although the precise timing of perinatal infection cannot be determined, modelling studies have suggested that infection early in pregnancy is rare and that most intrauterine infections are likely to have occurred towards the end of pregnancy [9]. Transmission also occurs intrapartum. Multiple studies have shown associations between younger age at start of ART and smaller size of the viral reservoir [10–16]. However, these studies are mostly small, have varying and often wide bounds around the time periods defined as early, and rarely include comparisons with later treated children. Moreover, only one report has described viral reservoir parameters from HIV-infected children living in sub-Saharan Africa where the HIV epidemic predominates [17].

Studies of the viral reservoir in HIV-infected children have additional methodological challenges related to the limited blood volumes that can be collected from children. Moreover, storage of viably-preserved PBMCs is costly and rare in studies undertaken in sub-Saharan Africa. Quantitation of cell-associated HIV-1 DNA is one marker of the viral reservoir that does not require large blood volumes or viably-preserved PBMCs. This marker is ideal when high sensitivity is desired i.e. detection of low levels. Its limitation is that it does not distinguish between viable and non-viable virus. Nevertheless, in a large study among early-treated adults, quantitation of cell-associated HIV-1 DNA emerged as the strongest predictor of clinically-relevant outcomes related to the size of the viral reservoir including time to viral rebound after ART withdrawal [6].

We developed an in-house, semi-nested real-time PCR assay to quantitate cell-associated HIV-1 DNA. The assay was designed with subtype C-specific primers and probes. Here we describe relationships between this marker of viral reservoir size and other factors including age at ART initiation, pre-treatment characteristics, and subsequent viral rebound, in a cohort of HIV-infected children treated as part of a clinical trial in Johannesburg, South Africa.

## Methods

### Study population

Stored samples were selected from children enrolled in an efavirenz-switch trial undertaken at Rahima Moosa Mother and Child Hospital in Johannesburg, South Africa (NCT01146873) [18, 19]. The trial randomized children who were suppressed (<50 HIV-1 RNA copies/ml) to remain on a ritonavir-boosted lopinavir (LPV/r)-based regimen or to switch to an efavirenz-based regimen. Other inclusion criteria included age older than 3 years and history of exposure to nevirapine for prevention of mother-to-child transmission (PMTCT). All children had met ART initiation criteria in place at the time. These included World Health Organization stage III or IV disease, CD4+ T-cell percentage <25 if younger than 12 months or <20 if older than 12 months, or recurrent (> 2/year) or prolonged (> 4 weeks) hospitalization for HIV-related complications. All children were initiated on a protease inhibitor-based regimen, mostly LPV/r. Stavudine and lamivudine were the most widely used backbone drugs. The trial was approved by the Institutional Review Boards of Columbia University and the University of the Witwatersrand. The child’s guardian provided signed informed consent.

For this analysis, we selected the 24 week post-randomization sample from all children in the trial who were 6 months of age or younger at the time of starting ART. One hundred and fifty-two children met these criteria and samples were stored on 132. In addition, we selected the same time point from a subset who had started ART 7–14 months of age. This subset consisted of those who had been enrolled in the control arm (LPV/r) in a prior nevirapine-switch trial [20] before enrolling in the efavirenz-switch trial. Twenty-nine children met these criteria and samples were stored on 26.

This sample of 158 children was intended to over-represent those who initiated ART at a young age. Plasma samples from the same time point were tested for anti-HIV-1 antibodies by enzyme immunoassay (GenescreenTM HIV1/2 version 2; Bio-rad) as previously described [21]. Consistent with manufacturer’s instructions, optical density (OD) readings below the standard were defined as “negative,” above the standard and above one OD unit were defined as “positive” and those with OD readings below one but above the standard were defined as “low-positive”.

Clinical and laboratory data collected as part of the efavirenz-switch trial was used to describe the population in terms of their pre-treatment characteristics, e.g. age at starting ART, pre-treatment viral load, CD4+ T-cell count/percentage, etc., early response to ART and characteristics at the time of HIV-1 DNA measurement. Children were followed until 48 weeks post-randomization as part of the trial. Thereafter they were enrolled in an observational study and followed with HIV RNA measurements at least every 6 months [19]. We included in this analysis follow-up data collected through 36 months after the index HIV-1 DNA measurement.

### Measurement of cell-associated HIV-1 DNA

We developed a semi-nested real-time quantitative hydrolysis probe (TaqMan) PCR assay (sn-qPCR) to detect and quantify total HIV-1 subtype C proviral DNA based on methods previously described [22, 23]. The assay was designed to target the *reverse transcriptase* (*RT*) gene of HIV-1 subtype C using all subtype C *pol* gene sequences available from the Los Alamos HIV-1 Database (http://www.hiv.lanl.gov).

Briefly, the sn-qPCR is a two-step PCR with the first round of amplification carried out using standard PCR on a conventional PCR machine and the following primers: forward primer 5ˈ-CAT TTC TTT GGA TGG GGT ATG A-3; reverse primer 5ˈ-CCT GTT CTC TGC CAA TTC TAA TTC TGC-3. The first round of PCR was allowed to proceed for 15 cycles only (i.e. halted in exponential phase). The total product of the first round PCR was subsequently used in the second round of PCR, a real-time hydrolysis probe-based PCR using a fluorescently-labelled TaqMan probe (5ˈ-FAM-AGC TGG ACT GTC AAT GA-MGB-3ˈ), one primer identical to the forward primer used in the first round PCR and a second reverse primer (reverse primer2: 5ˈ-TTG CCC AGT TTA ATT TTC CCA CTA-3ˈ) designed to bind ‘deeper’ within the amplicon. The second round PCR was carried out for the standard cycle numbers used in real-time PCR (40 cycles). The first round PCR was set up in a 12 ¼l final volume and subsequently diluted 1:8.3 to a 100 μl final volume in the second round qPCR.

The numbers of HIV-1 proviral DNA copies were determined using the standard curve method. To construct the standard curve, known copies of linearized p8MJ4 plasmid DNA, cloned with a subtype C gag-pol gene, were serially-diluted and amplified in a background of HIV-1-negative human gDNA (the same amount used in the experimental wells) and subjected to the same cycling conditions described above. To minimize the possibility of cross-contamination all reactions were set up using a 8-well PCR tube strip format with standard curves being run concurrently on their own 8-well strip.

**Fig 1:**
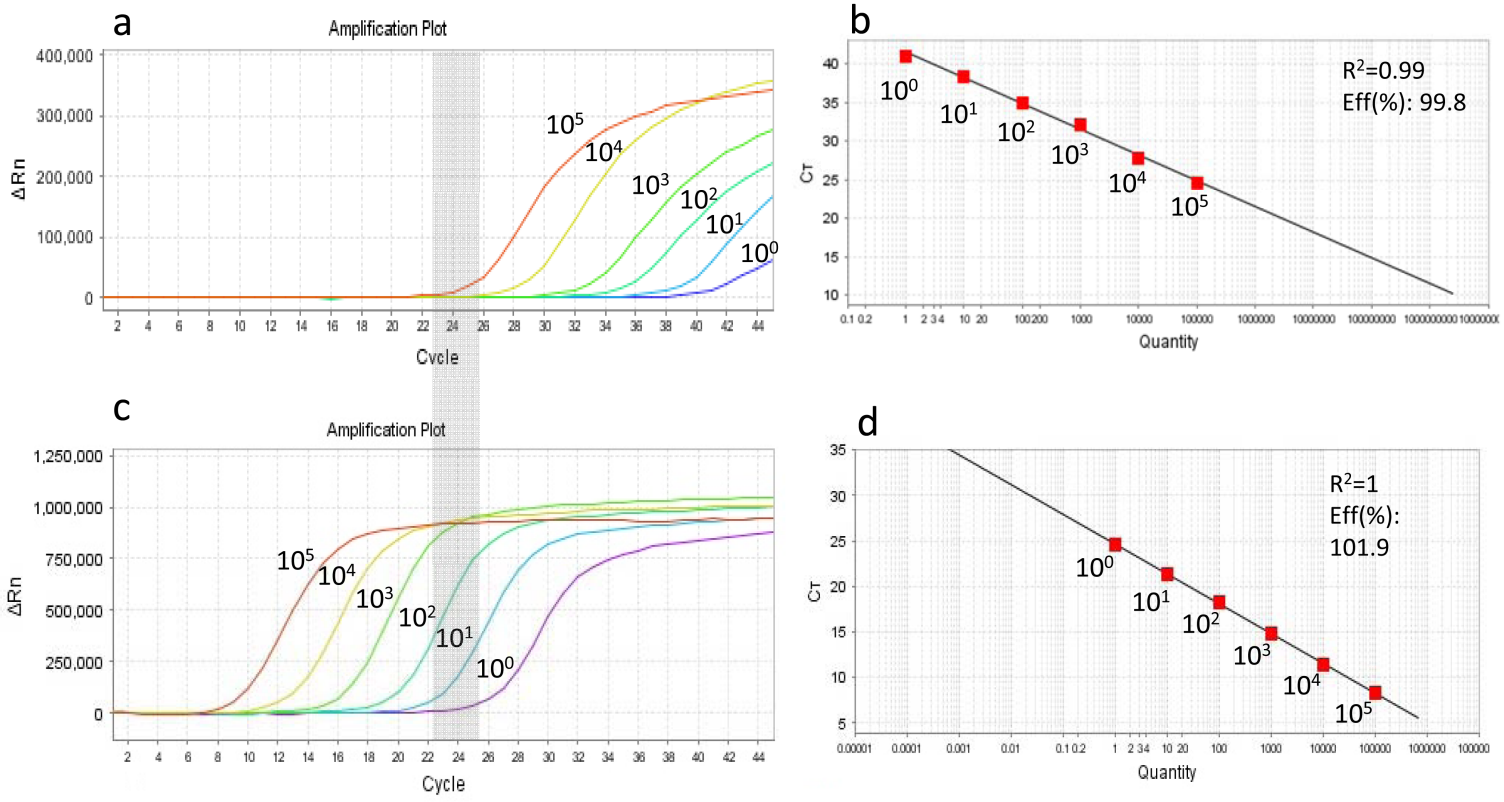
Comparison of *reverse transcriptase*-based single round qPCR (a and b) and sn-qPCR (c and d) assays designed to quantify subtype C HIV-1 proviral DNA. Amplification curves (a and c) of ten-fold diluted plasmid (p8MJ4) DNA harboring subtype C HIV-1 gag-pol gene and standard curves generated (b and d) from respective curves. Grey zone shows that the cycle threshold (C_T_) region where 10^5^ plasmid DNA copies are amplified in the single round qPCR (a) is equivalent to the C_T_ region where a single copy (10°) is amplified using the sn-qPCR assay. Eff = PCR efficiency.

We compared serial plasmid dilutions that were amplified either by our in-house sn-qPCR assay or a single-round qPCR using the same forward primer, reverse primer2 and probe (Fig 1). Although both the semi-nested as well as the single round assays can detect 1 single copy, the cycle threshold (C_T_) where 10^5^ copies of plasmid DNA is detected in the single round PCR is equivalent to the C_T_ region where a single copy is detected in the sn-qPCR (grey zones in Fig 1a and 1c). Thus a shift in C_T_ from approximately 38 (single round PCR) to 25 (sn-qPCR) for single copy amplification allows for considerably more confidence in the lower copy number calls using sn-qPCR. Fig 1b and 1d show the corresponding standard curves and both have R^2^ values > 0.99 and PCR efficiencies > 99%. The assay was highly efficient at amplification of plasmid DNA down to a single copy (10°) as shown in Fig 1c and 1d.

Genomic DNA was extracted from 158 stored buffy coats (QIAamp DNA blood kit; Qiagen, Düsseldorf, Germany) selected for this analysis out of which 151 samples yielded sufficient DNA for assaying (minimum cut-off of 400ng [6x10^4^ leukocytes]). Where sufficient DNA was available, samples were tested in triplicate (3 wells) with 875ng DNA/well (equivalent to 3.98x10^5^ leukocytes). In cases where there was insufficient DNA to perform three replicates at that concentration, we tested all DNA available for that sample and if a positive result was attained we calculated the DNA copies according to input gDNA (6/151 two replicates and 3/151 one replicate). If we failed to get a result on the lower input DNA we intended to exclude that sample from the analysis since we could not conclusively call it “undetected” due to the lower input of DNA. However, none of the nine had to be excluded for this reason. Although the assay was capable of detecting a copy number of 1, since we were only assaying 2625ng across three wells for most samples, a single copy detected in 2625ng equates to 3 copies/10^6^cells, making this the limit of detection of the assay implemented in this study.

### Statistical methods

HIV-1 DNA copies were expressed on the log_10_ scale with undetectable results recoded as 1 copy/10^6^ cells = 0 log_10_ copies. The distributions of DNA log_10_copies were plotted using histograms and medians and interquartile ranges (IQR) were calculated. Box-whisker plots were generated by key covariates. Analysis of variance (ANOVA) and pairwise t-tests were used to test for differences between groups. Spearman Rank order correlations (rho) were calculated to describe associations on the continuous scale. The non-parametric smoothing method Locally Weighted Scatterplot Smoothing (LOWESS) was used to inspect for non-linear associations. Chi-squared tests were used to compare categorical variables between groups. To investigate whether there was an association between HIV-1 DNA levels and subsequent viral rebound/treatment failure on ART, we divided the cohort above and below the median of 55 HIV-1 DNA copies/10^6^ cells. Kaplan-Meier methods were used to examine time to viral rebound/failure on ART >50, >400 and >1000 copies/ml over time up to 3 years after DNA measurement in these two groups. P-values were calculated using log-rank tests. Multivariable linear regression and Cox Proportional Hazards models were used to investigate independent effects of the predictors. Analysis was conducted using SAS software (Cary, NC).

## Results

### Study participants

Of 151 children with HIV-1 DNA measured, 5 children had HIV-1 RNA >50 copies/ml at the same visit and were excluded. The 146 HIV-infected children suppressed on ART at the time of measurement had been initiated on ART between 6 weeks and 14 months of age (median 4.5 months, IQR: 3.0–5.8), were now a median of 4.6 years old (IQR: 3.9–5.2) and had been treated for a median of 4.3 years (IQR 3.7–4.8) when cell-associated HIV-1 DNA was measured. Pre-treatment characteristics and profile at the time of measurement are shown in Table 1. HIV-1 DNA could not be detected in three samples (2.1%) and the minimum observed level of HIV-1 DNA was 3 copies/10^6^ cells. The maximum was 1077, median 55, and IQR 25 to 118 copies/10^6^ cells.

**Table 1:** Characteristics of 146 treated HIV-infected children in Johannesburg, South Africa, at the time of measurement of cell-associated HIV-1 DNA (current time) and at the time of antiretroviral therapy (ART) initiation.

	N^a^ (%)	Median (IQR)
		[min, max]
**Child sex**		
Male	64 (43.8)	
Female	82 (56.2)	
**Age at ART start in months**	146	4.47 (2.96–5.76)
		[0.79, 13.75]
**Current age in years**	146	4.58 (3.90–5.16)
		[3.53, 7.54]
**Duration of ART until measurement in years**	146	4.26 (3.65–4.76)
		[2.93, 6.40]
**Current ART regimen**		
Efavirenz-based	76 (52.1)	
Lopinavir/ritonavir-based	70 (47.9)	
**Current CD4 count in cells/mm**^**3**^	140	1221 (977–1659)
		[493, 3630]
**Current CD4 percentage**	140	35.4 (30.2–40.6)
		[17.5, 56.9]
**Pre-ART CD4 count in cell/mm3**	137	1396 (755–1989)
		[21, 3677]
**Pre-ART CD4 percentage**	139	23.4 (16.1–30.2)
		[2.9, 80.0]
**Pre-ART HIV-1 RNA**^b^ **copies/ml**	127	750,000 (270,000–750,000)
		[73, 73,000,000]
<100,000	14 (11.0)	
100,000–749,999	36 (28.4)	
>750,000	77 (60.6)	

^a^ Denominators are as shown and when less than 146 are explained by missing data

^b^ Assays to quantitate HIV-1 RNA in clinical use at the time did not consistently quantitate >750,000 copies/ml

### Age at ART initiation

**Fig 2:**
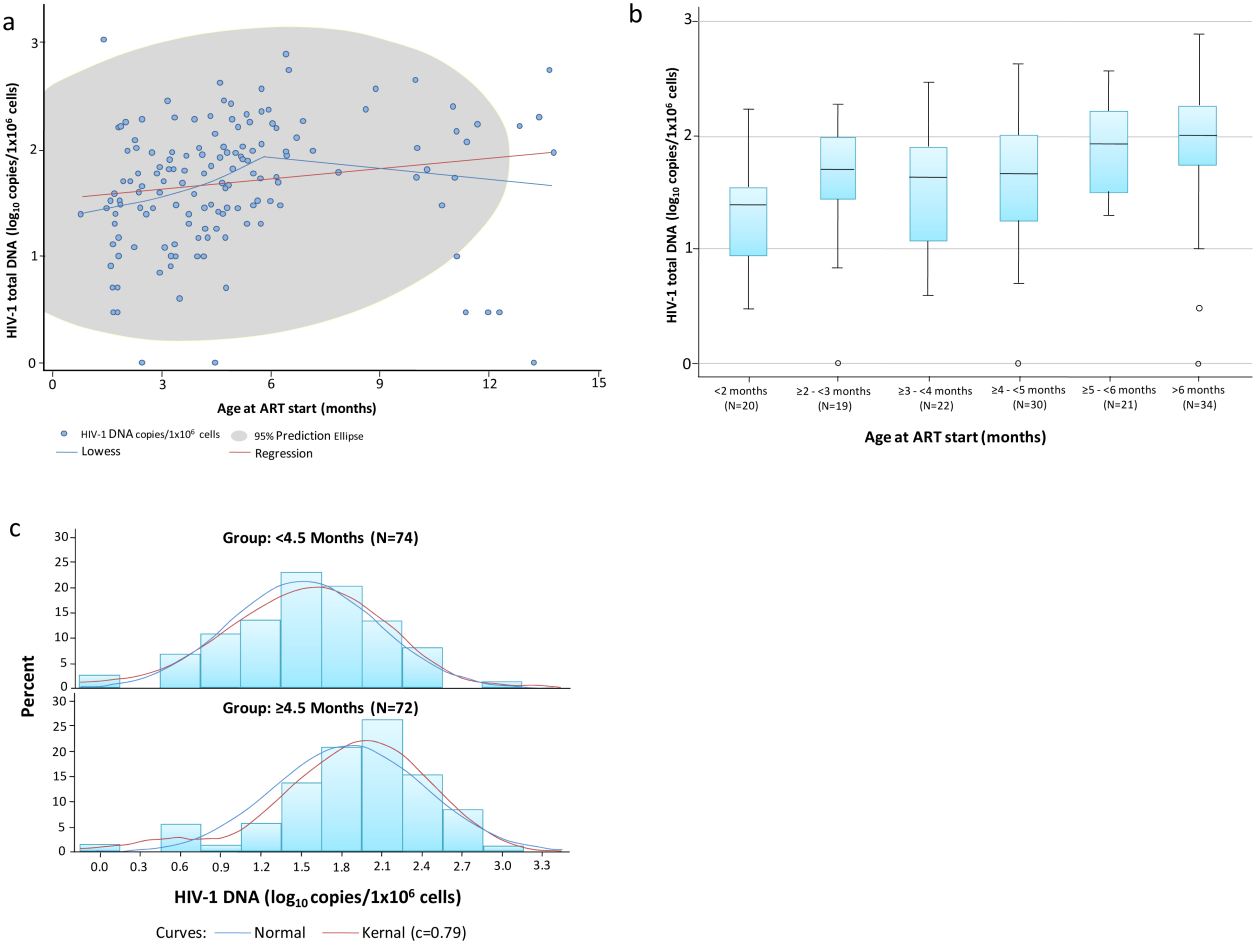
Associations between age at starting ART and cell-associated HIV-1 DNA levels after a median of 4.3 years of treatment among 146 HIV-infected children. a: Scatterplot of HIV-1 DNA log_10_ copies/10^6^ cells (Y-axis) by age at ART start in months (x-axis). LOcally WEighted Scatter-plot Smoother (LOWESS) and linear regression prediction lines are shown in blue and red, respectively; and the grey area shows the 95% prediction elipse. b: Box and whisker plot of HIV-1 DNA log_10_ copies/10^6^ cells by age at ART start groups; c: Histograms and estimated normal and kernel distributions of HIV-1 DNA log_10_ copies/10^6^ cells among those who started ART <4.5 and ≥4.5 months of age.

A significant but weak positive correlation was observed between HIV-1 DNA log_10_ copies/10^6^ cells and age at ART start in months (rho = 0.327, p < .0001). A non-parametric smoothing of the relationship (LOWESS) suggested that the relationship was linear through 5 months without further association thereafter (Fig 2a). A box and whisker plot of HIV-1 DNA log_10_ copies/10^6^ cells and age at ART start in grouped month categories indicated that ART initiation at a younger age was associated with lower levels of HIV-1 DNA (Fig 2b). ART started at ≥5 months had the highest levels of HIV-1 DNA (median 1.98, IQR 1.69–2.25 log_10_ copies/10^6^), compared to ART started 2–4 months (median 1.68, IQR 1.26–1.97, p = 0.02) and started under 2 months of age (median 1.4, IQR 0.95–1.55 p = 0.002). Examining the distribution of HIV-1 DNA stratified into two groups above and below the median age of starting ART revealed higher HIV-1 DNA levels in children who started ART ≥4.5 months (median = 1.96, IQR 1.52–2.25) compared to those starting ART <4.5 months of age (median 1.52, IQR 1.11–1.9, p = 0.0003) (Fig 2c). Two of the three children with undetectable HIV-1 DNA had started ART below the median age of the group.

### Other predictors

**Fig 3:**
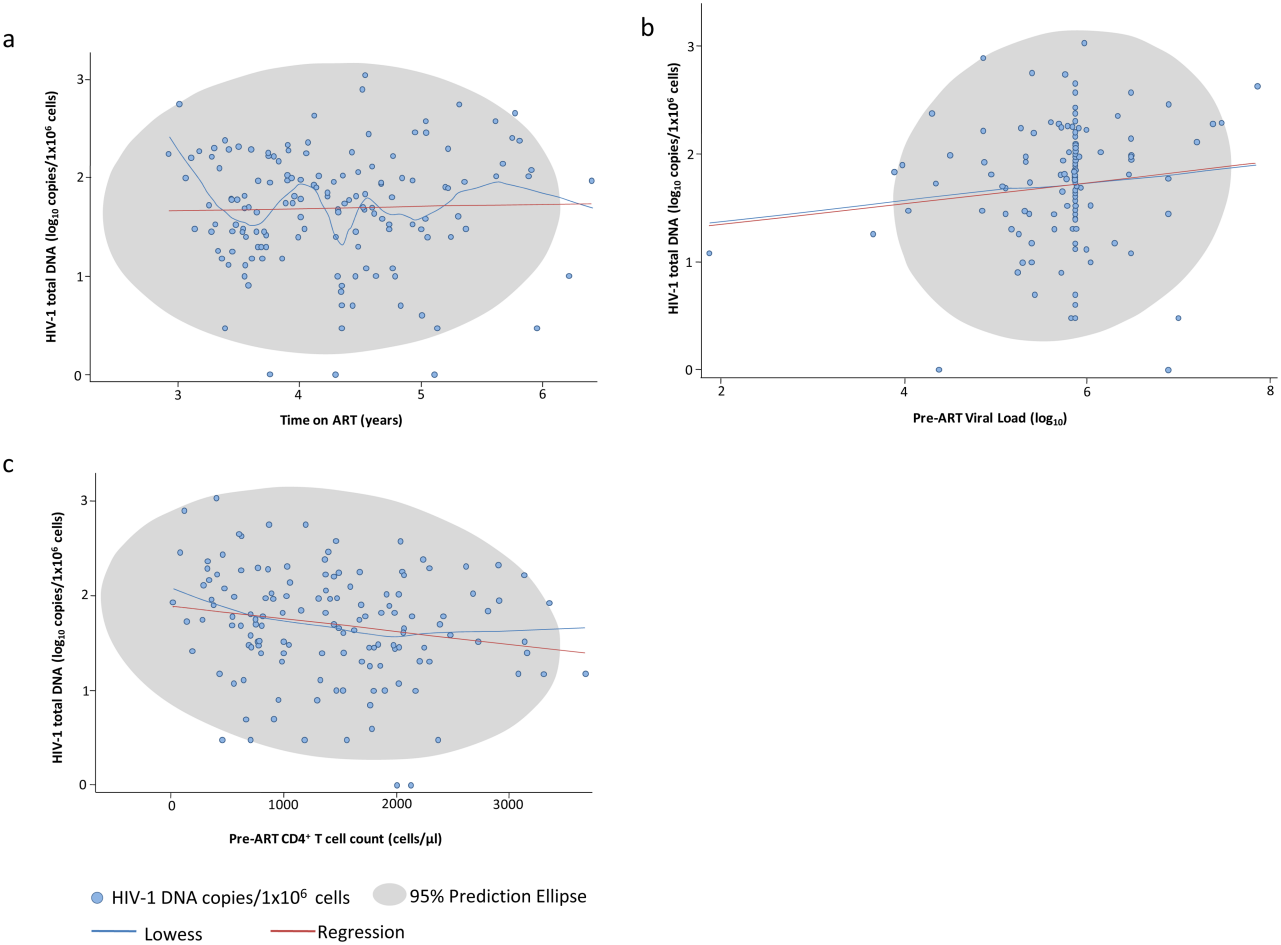
Scatterplots showing associations between HIV-1 DNA log_10_ copies/10^6^ cells and a: Time on ART in years; b: Pre-ART HIV-1 RNA copies/ml and c: Pre-ART CD4 count in cells/mm3. LOcally WEighted Scatter-plot Smoother (LOWESS) and linear regression prediction lines shown in blue and red, respectively.; grey area shows the 95% prediction elipse.

**Fig 4:**
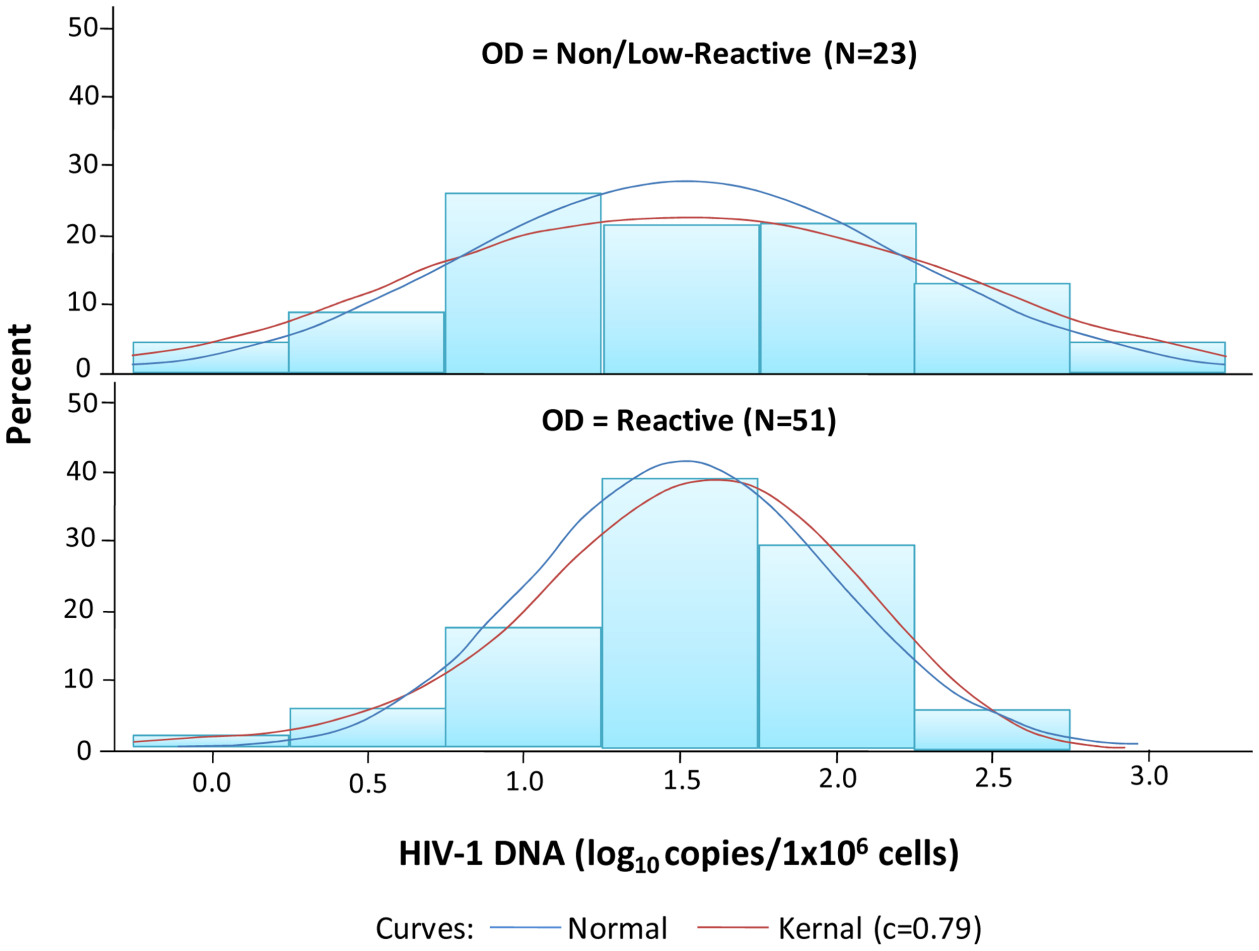
Histograms and estimated normal and kernel distributions of HIV-1 DNA log_10_ copies/10^6^ cells by reactive or non-reactive/low HIV-1 antibody results among 74 HIV-infected children who initiated ART < 4.5 months of age.

Duration of ART to the time of measurement was not associated with HIV-1 DNA levels (r = 0.029; p = 0.73) (Fig 3a) nor was the closely related variable of child age (S1 Fig). There was no association between HIV-1 DNA levels and child sex, current ART regimen, or current CD4 T cell count or percentage (S2, S3, S4 and S5 Figs, Figs 3 and 4).

Lower pre-ART CD4+ T-cell count was significantly associated with higher HIV-1 DNA levels (rho = -0.197, p = 0.02) (Fig 3c). Stratifying into pre-ART CD4+ T-cell count categories, higher HIV-1 DNA levels were observed in those with a pre-ART CD4 count <500 cells/mm^3^ (median = 2.09, IQR 1.74–2.36) than in those with pre-ART CD4 counts 500–999 and >1000 cells/mm^3^ (median = 1.69, IQR 1.4–1.99, p = 0.09 and median = 1.68, IQR 1.3–2.02, p = 0.02, respectively). Pre-ART CD4+ T cell percentage followed the same pattern but associations were weaker and non-significant. Higher pre-ART HIV-1 RNA levels showed a non-significant trend towards being associated with higher HIV-1 DNA levels (rho = 0.122, p = 0.17) (Fig 3b).

To examine whether speed of initial HIV-1 RNA decline predicted later HIV-1 DNA levels, we compared children who had achieved HIV-1 RNA <400 copies/ml by 6 months after ART start (53.4%) to those who achieved suppression only later. There was no discernable difference in the HIV-1 DNA levels by this marker of speed of initial viral decline (S6 Fig).

The strong association between age at ART start was unchanged with the inclusion in a multivariable model of pre-ART CD4+ T-cell count, pre-ART viral load, or duration of treatment.

### Association with HIV-1 antibody test

A non-reactive or low reactive HIV-1 antibody test was observed at the same time as the HIV-1 DNA measurement in 23/74 (31.1%) children who started ART <4.5 months compared to 4/69 (5.8%) children who started ≥4.5 months. We therefore restricted analysis of the association between HIV-1 antibody and HIV-1 DNA levels to children who started ART <4.5 months. The median HIV-1 DNA levels were almost identical in those with non-reactive/low vs. reactive HIV-1 antibody results (33 copies/10^6^ cells) but the variance was larger in those with non-reactive/low compared to reactive antibody results (0.515 vs. 0.235 log_10_ copies, respectively, p = 0.02) (Fig 4).

### Time to HIV-1 RNA rebound/failure on ART

Using Kaplan-Meier methods, time to HIV-1 RNA rebound >50 copies/ml, >400 copies/ml and >1000 copies/ml up to 36 months after the index HIV-1 DNA measurement was significantly faster for children with HIV-1 DNA >55 copies/10^6^ cells compared to those with HIV-1 DNA levels ≤55 copies/10^6^ cells (Table 2). When children were stratified by age at ART initiation, stronger associations between HIV-1 DNA levels (each log_10_ increase) and subsequent HIV-1 RNA rebound >50 copies/ml on ART (relative hazard (RH) = 2.908; 95% CI: 1.627–5.200) were observed in the cohort who had started ART <4.5 months compared to the cohort who started ART ≥ 4.5 months (RH = 1.308; 95% CI: 0.745–2.294) (Table 2). However, the interaction term in the Cox model was not statistically significant. When considered on its own, starting ART ≥ 4.5 months of age was associated with increased likelihood of HIV-1 RNA rebound >50 copies/ml on ART compared to starting ART earlier (RH = 1.55; 95% CI: 1.022–2.354). In a multivariable Cox model in the full cohort, cell-associated HIV-1 DNA (each log_10_ increase) remained a strong predictor of subsequent rebound >50 copies/ml on ART (RH = 2.146; 95% CI: 1.427–3.227) after adjusted for age at starting ART but age at starting ART was no longer associated with subsequent viral rebound once HIV-1 DNA was in the model. Inclusion of other covariates such as pre-treatment CD4+ T-cell count, pre-treatment HIV-1 RNA and duration of treatment did not influence the results.

**Table 2:** Kaplan-Meier probability of HIV-1 RNA rebound during follow-up on treatment after measurement of cell-associated HIV-1 DNA.

	Cell-associated HIV-1 DNA^a^			
	≤55 copies/10^6^ cells	>55 copies/10^6^ cells	Log-rank p-value	
N	74	71		
				Hazard Ratio from Cox model comparing HIV-1 DNA >55 to ≤ 55 copies/10^6^ cells
Probability of HIV-1 RNA >___ by ____ months (N with event)				
**>50 copies/ml**				
12 months	0.1020 (7)	0.1699 (10)		
24 months	0.3681 (24)	0.4878 (28)		
36 months	0.4629 (30)	0.7008 (40)	0.0006	2.07(95% CI: 1.352–3.167)
**>400 copies/ml**				
12 months	0.0137 (1)	0.0837 (5)		
24 months	0.1383 (9)	0.2274 (13)		
36 months	0.1696 (11)	0.3177 (18)	0.0021	2.641 (95%CI: 1.389–5.022)
**>1000 copies/ml**				
12 months	0.0137 (1)	0.0837 (5)		
24 months	0.0915 (6)	0.1915 (11)		
36 months	0.1072 (7)	0.2458 (14)	0.0187	2.526 (95%CI: 1.134–5.626)

^a^ One child had no follow-up after HIV-1 DNA measurement and was excluded from this analysis.

## Discussion

We report here a gradient between younger age at ART initiation and lower levels of cell-associated HIV-1 DNA measured in children suppressed on ART for just over 4 years. The gradient of benefit extended to starting ART up until around 6 months of age without a discernable benefit thereafter. The Feibig classification system cannot be applied to perinatal infection. However, in adult studies, treatment within 3–6 months of HIV-1 diagnosis, is often included as a treatment category for acute infection [1–3]. Our results are consistent with other studies in children but provide greater clarification of the effects of early ART in narrower age intervals and in comparison to comparable control children initiating ART only slightly later [10–17]. Our assay was developed specifically for subtype C virus and targeted *pol* because of the highly-conserved regions within this gene. Nevertheless, the primers do not overlap with any major NNRTI or NRTI mutations, and is therefore suitable for treated patients.

We did not observe associations between cell-associated HIV-1 DNA and duration of ART. This may be due to the cross-sectional design and only limited heterogeneity in cohort of duration of ART. In addition, since we measured HIV-1 DNA after more than 4 years of ART the size of the viral reservoir may have stabilized. Generally, the most marked declines in HIV-1 DNA levels are within the first two years of treatment [13]. Associations between pre-treatment characteristics including CD4 T cell count and HIV-1 RNA quantity were consistent with expectations that pre-treatment disease severity would predict a larger persisting viral reservoir. Higher concentrations of lymphocytes and different profiles of lymphocyte subsets in neonates and infants compared to adults is one of the hypothesized reasons for potentially favorable outcomes in early-treated infants [24, 25]. Surprisingly, CD4+ T-cell count was a stronger predictor than CD4 T cell percentage which is the usually preferred parameter used in clinical management of HIV-1-infected infants and young children. These results indicate that, in this instance, pre-treatment CD4+ T-cell count is more informative than percentage.

The median concentrations of cell-associated HIV-1 DNA did not differ between those with and without positive HIV-1 antibody results. We confined this analysis to children who had initiated ART under 4.5 months of age as we have previously reported that negative or low antibody test results were fairly common in this age group but rarely observed in children who started ART after 4 months of age as have others [21, 26]. We expected to observe that low/negative antibody results would correlate with lower viral reservoirs but the data did not support this. We hypothesize that the third generation antibody assay that we used is too crude a measure to discern how the viral reservoir might influence persisting HIV-1 antibodies. The greater variability in the size of the viral reservoir in those children with negative/ low antibody results is intriguing and should be further investigated longitudinally and with finer resolution of antibody isotypes and antigen specificity.

A single cross-sectional measure of cell-associated HIV-1 DNA among children suppressed on ART was a strong predictor of later HIV-1 RNA rebounds in plasma while children remained on their ART regimen. Consistent adherence with lifelong ART is a challenge for all HIV-infected individuals but has unique poignancy for growing children and their caregivers. Fluctuating adherence over time is the most likely explanation for the viral rebounds observed. Although believable measures of adherence are difficult to attain, we have no reason to expect that adherence differed between children with larger vs. smaller viral reservoirs. Thus our results suggest that children with smaller viral reservoirs may be somewhat protected from treatment failure with fluctuating adherence. There are differing opinions about the value of ART withdrawal in early-treated individuals once suppressed given that the rate of post-treatment viral control for reasonable lengths of time is achieved in only a small minority. However, unplanned, unsupervised and usually unnoticed gaps in adherence are an almost inevitable pattern in at least some patients. Our findings suggest that even through pessimism about the capacity of early treatment on its own to achieve remission may be valid, a smaller viral reservoir may provide some level of protection against treatment failure in the almost inevitable event of periods of sub-optimal adherence.

Our study had several limitations. Viably-preserved PBMCs were not available for this cohort. As a result HIV-1 DNA levels may be under-estimated compared to what may have been observed if PBMCs and especially if isolated CD4+ PBMCs had been available. A single marker was used to estimate the size of the viral reservoir and further studies characterizing viral integration, viability, replication competence and other parameters will most certainly be important. Despite the large size of the cohort in comparison to previous studies, numbers in some sub-groups is small. Moreover, the relatively weak size of the association between HIV-1 DNA levels and age at starting ART and the wide variability indicate that age at treatment start is only one factor among many influencing the size of the viral reservoir. Many parameters, including the size of the HIV-1 DNA pool prior to starting ART a factor shown to be associated with later size of the reservoir [12, 27], were not available. HIV-1 DNA was measured at a single time point several years after ART initiation. Longitudinal investigation of the viral reservoir profile during the first two years after ART is initiated will be crucial to further our understanding of how (and when) to intervene.

## Conclusion

We provide strong evidence of an association between ART initiation within the first few months of life and a reduced size of the viral reservoir several years later in children suppressed on ART. We also demonstrate that a smaller viral reservoir once suppressed on ART is associated with lower likelihood of viral RNA rebounds over time among children on treatment. The results provide additional evidence of the benefits of prompt diagnosis and early ART initiation in newborns and infants.

## Supporting information

S1 Fig Scatterplot of HIV-1 DNA log10 copies/106 cells (Y-axis) by age of child when tested. (TIF)

S2 Fig Histograms and estimated normal and kernel distributions of HIV-1 DNA log10 copies/106 cells by child sex: F = Female top panel M = Male lower panel. (TIF)

S3 Fig Histograms and estimated normal and kernel distributions of HIV-1 DNA log10 copies/106 cells by current treatment regimen: EFV = Efavirenz-based and LPV/r = ritonavir-boosted lopinavir-based regimen. (TIF)

S4 Fig Scatterplot of HIV-1 DNA log10 copies/106 cells by current CD4 count. (TIF)

S5 Fig Scatterplot of HIV-1 DNA log10 copies/106 cells by current CD4 percentage. (TIF)

S6 Fig Histograms and estimated normal and kernel distributions of HIV-1 DNA log10 copies/106 cells by whether or not viral suppression (<400 copies/ml) was attained by 6 months after starting ART. No = suppression was not attained within 6 months of starting ART. Yes = Suppression was attained at or before 6 months after starting ART.
(TIF)
