# Supplementary figures and images for: Age at antiretroviral therapy initiation and cell-associated HIV-1 DNA levels in HIV-1-infected children
Source: PLoS One. 2018 Apr 12;13(4):e0195514. doi: 10.1371/journal.pone.0195514 (PMC5896970; doi:10.1371/journal.pone.0195514)

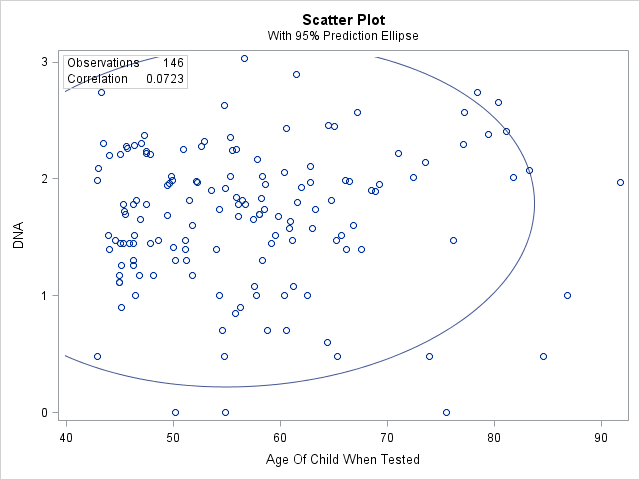

Supplement: S1 Fig — (TIF) [file pone.0195514.s001.tif]

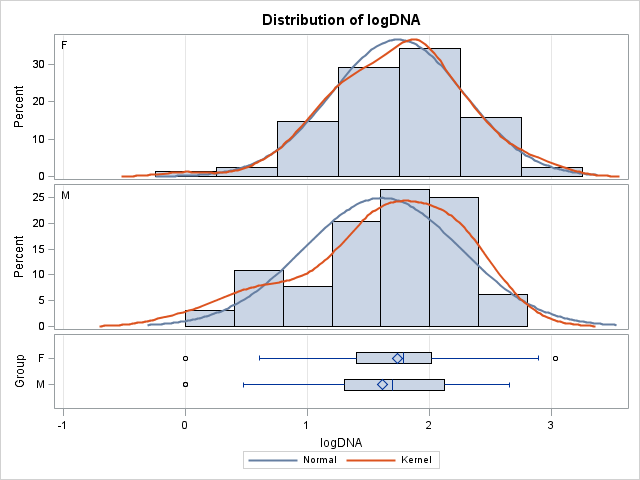

Supplement: S2 Fig — (TIF) [file pone.0195514.s002.tif]

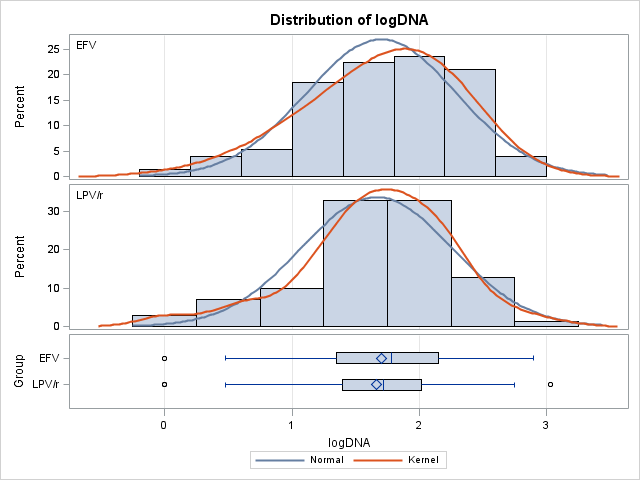

Supplement: S3 Fig — (TIF) [file pone.0195514.s003.tif]

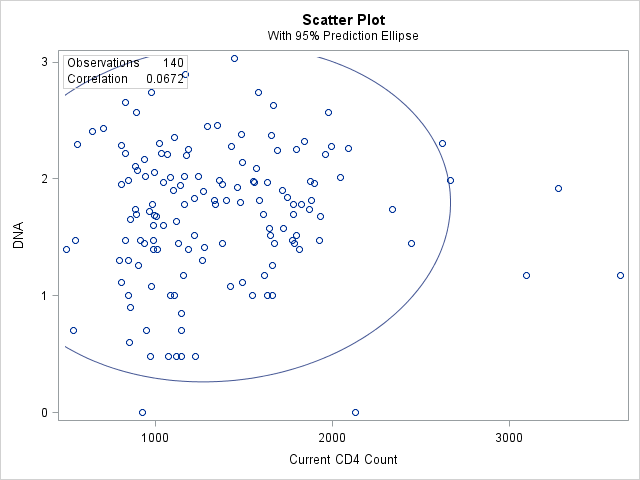

Supplement: S4 Fig — (TIF) [file pone.0195514.s004.tif]

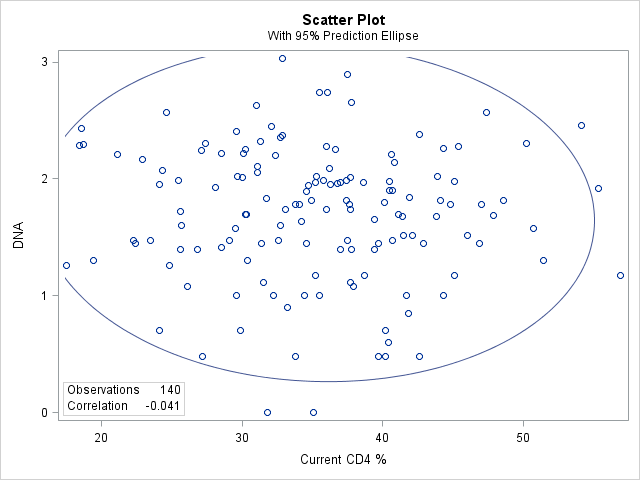

Supplement: S5 Fig — (TIF) [file pone.0195514.s005.tif]

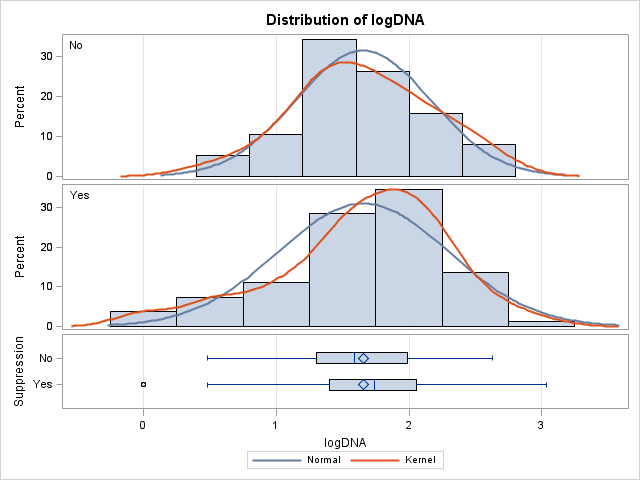

Supplement: S6 Fig — No = suppression was not attained within 6 months of starting ART. Yes = Suppression was attained at or before 6 months after starting ART. (TIF) [file pone.0195514.s006.tif]
